# Malignant Mucosal Melanoma of the Paranasal Sinuses

**DOI:** 10.7759/cureus.93257

**Published:** 2025-09-26

**Authors:** Naglaa Elsayed, Aseil Mahboob, Aisha Almassawi

**Affiliations:** 1 Radiological Sciences, King Abdulaziz University, Jeddah, SAU; 2 Diagnostic Radiology, Faculty of Medicine, Cairo University, Cairo, EGY; 3 Nursing, King Abdullah Medical City, Jeddah, SAU

**Keywords:** ct, melanoma, mri, mucosal, mucosal melanoma, nasal, paranasal sinuses, sinus

## Abstract

Sinonasal melanoma is a rare malignant tumor arising from the melanocytes of the paranasal sinuses. It has local and distant metastasis. This study reports a case of sinonasal melanoma at King Abdullah Medical Center. A 74-year-old male patient presented with unilateral nasal obstruction and epistaxis. A contrast-enhanced CT scan of the brain and paranasal sinuses showed findings suggestive of invasive fungal sinusitis. Following this finding, the patient was referred for an MRI scan, which confirmed the aggressive nature of the mass. Subsequently, a biopsy was taken from the mass, which proved sinonasal melanoma. The patient underwent surgical resection of the lesion. Histopathology confirmed the diagnosis of sinonasal melanoma. Unfortunately, the patient passed away one week after the operation.

## Introduction

Sinonasal mucosal melanoma (SNMM) is one of the rare, highly aggressive tumors often diagnosed at an advanced stage [1]. First described in 1856, mucosal melanoma originates from melanocytes of the mucosal membranes of the nasal cavity, sinuses, oral cavity, lips, and pharynx, in addition to other mucosal surfaces of the genital and anorectal regions [2]. Risk factors include pre-existing sinonasal melanosis; however, the exact environmental carcinogen is not known [1]. Mucosal melanoma of the head and neck represents about half of all mucosal melanomas, and 80% of them are SNMM [3].

SNMM is usually diagnosed at an advanced age, around 70 years [4]. Due to their hidden location and lack of clear early symptoms, malignant melanomas are often diagnosed late [5]. Sinonasal melanoma has a high ability to send metastases, both local, usually to the cervical lymph nodes, and distant, commonly affecting the lungs and liver [6].

No specific symptoms point to the presence of SNMM. Patients are usually presented with nonspecific upper respiratory tract symptoms, mainly unilateral nasal obstruction and epistaxis. Some cases present with facial morphological changes due to local tumor invasion, in addition to local pain and frontal headache. Diplopia, epiphora, and ptosis are also common presentations in advanced stages due to local effects on the orbital structures. However, the relationship between SNMM images and clinical features is still unclear [7-10].

Sinonasal melanoma has a poor prognosis, with five-year survival rates between 20% and 55% [11,12], with a greater than 50% rate of recurrence [1].

CT and MRI are essential for diagnosing and staging of SNMM, detecting local invasion of the adjacent structures, and evaluating possible metastasis [13]. Wide transnasal-endoscopic tumor resection, followed by postoperative radiation therapy, remains the primary approach for managing SNMM [14].

## Case presentation

The patient was a 74-year-old male admitted to King Abdullah Medical Center, known to have diabetes mellitus and hypertension. He was suffering from right-sided nasal obstruction for two months, associated with headache and epistaxis, which responded to conservative management, and then became progressive. No history of ophthalmoplegia, diplopia, or decreased vision was present. He worked in a gypsum factory for the last four years and was a non-smoker. No family history of malignancy was noted. Physical examination showed a right fungating nasal mass occupying the right nasal cavity and extending to the nasopharynx with fragile mucosa. No neck masses were present. Ophthalmology examination revealed left mild to moderate proptosis with lateral inferior temporal dystopia. He was referred to the high center for further management. Imaging was requested for further investigation.

Imaging

**Figure 1:**
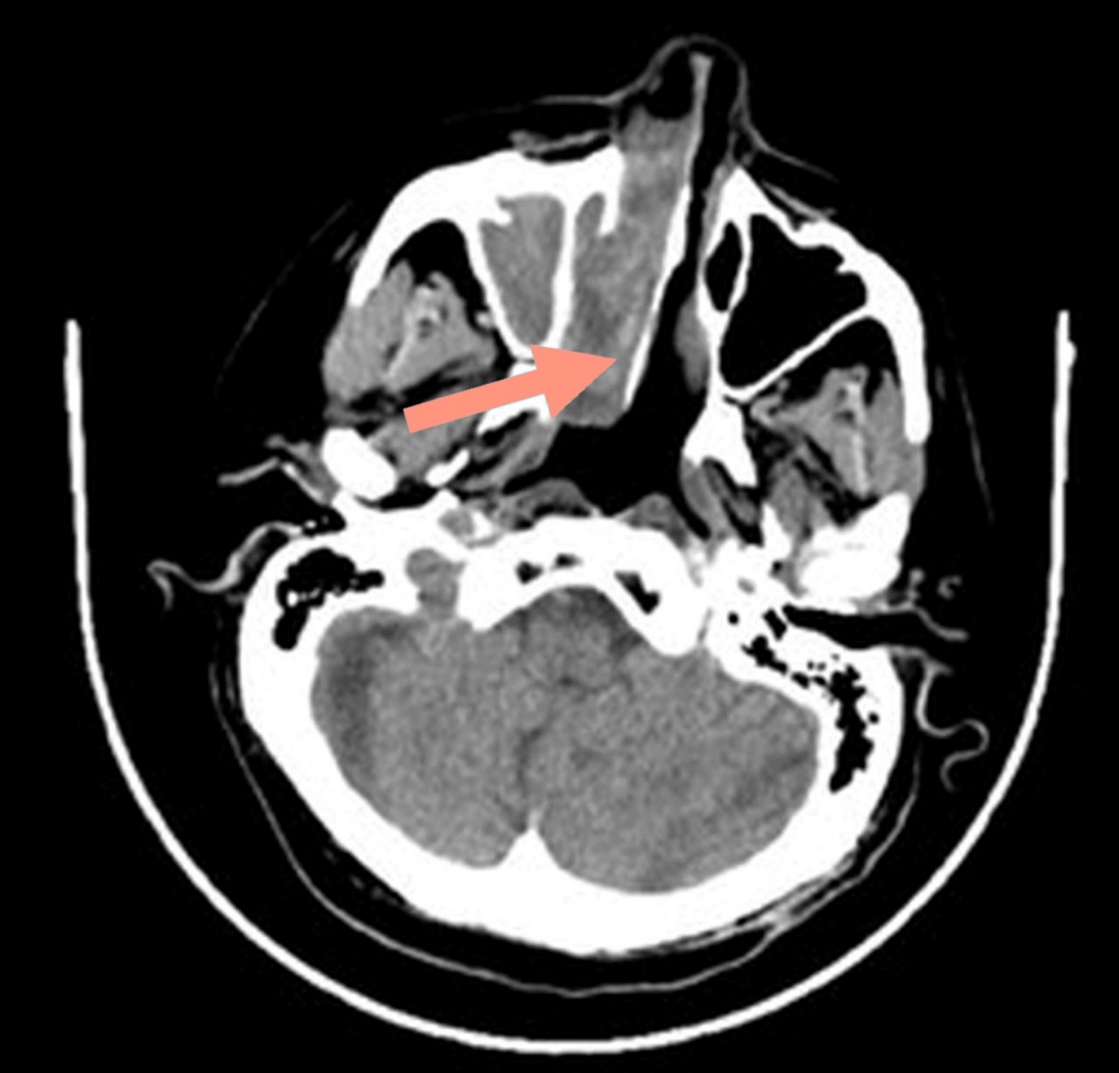
Axial CT with contrast showing right nasal cavity mass extending into the nasopharynx (arrow).

**Figure 2:**
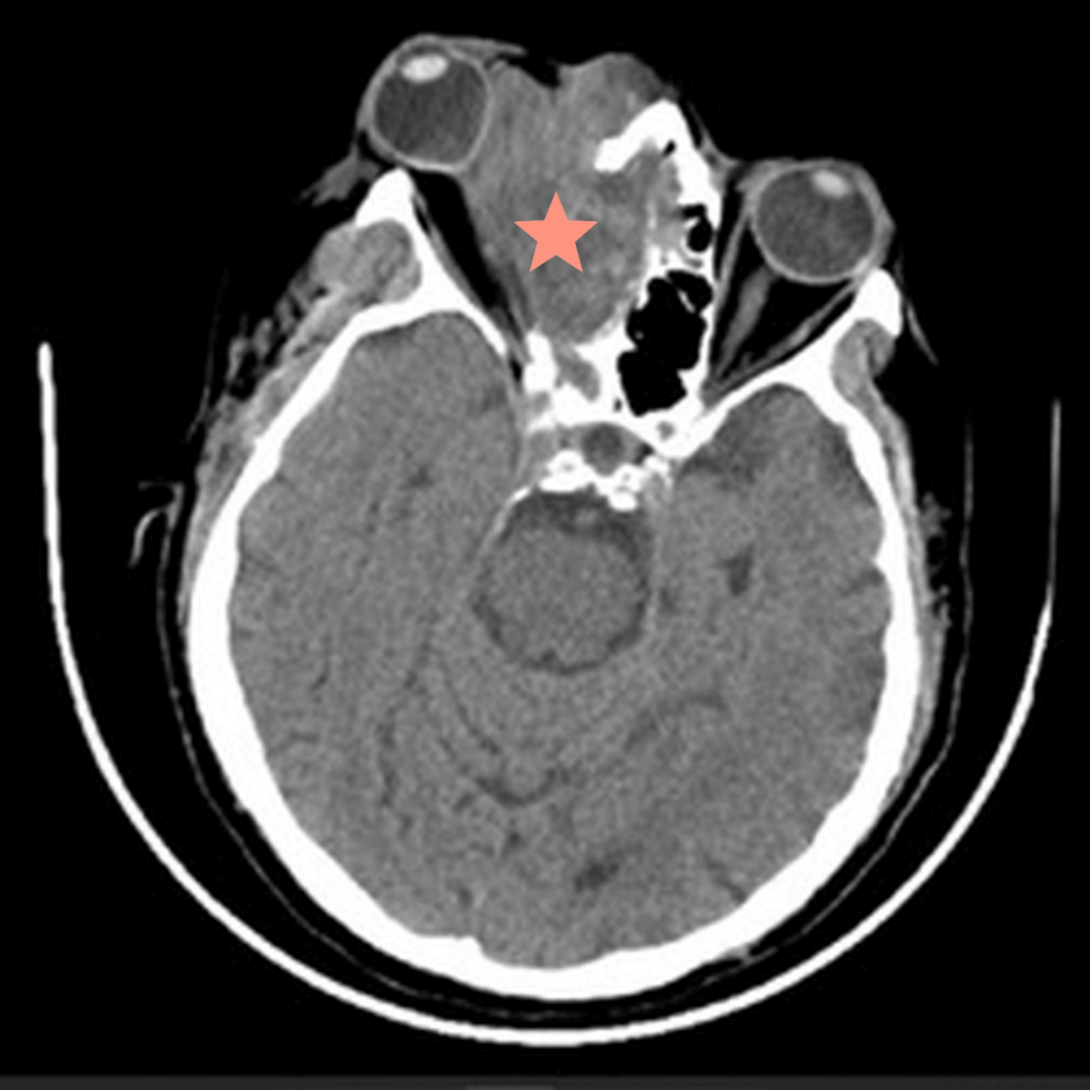
Axial brain CT showing extension of the mass into the right orbital cavity (star).

**Figure 3:**
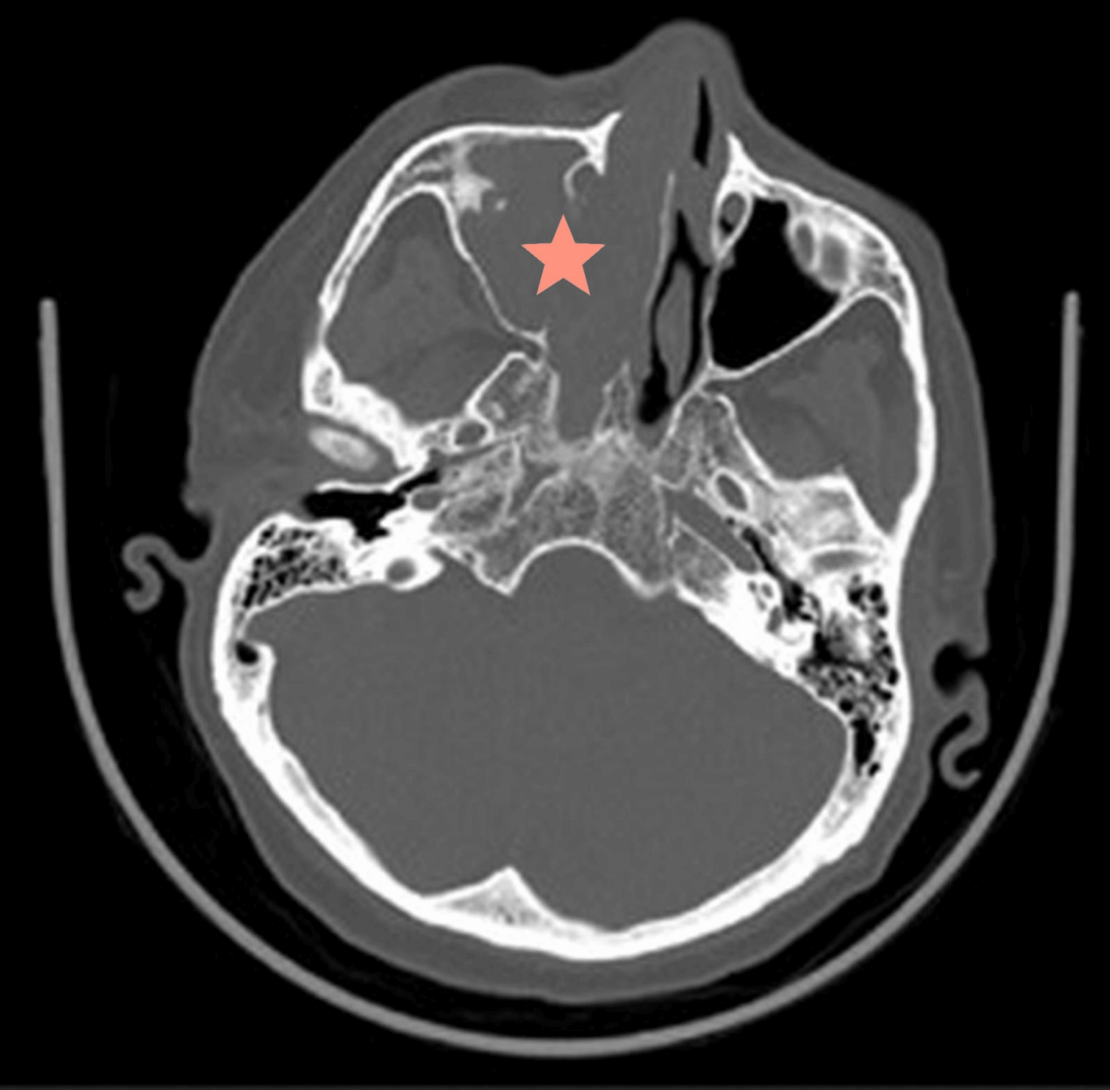
Axial brain CT bone window showing bone destruction due to aggressive nature of the tumor (star).

CT with contrast findings were related to aggressive fungal sinusitis in a background of antrochoanal polyp with secondary sinusitis. Figure 1 shows a mass arising from the right nasal cavity and extending to the nasopharynx. Figure 2 shows infiltration of the right orbit structures by the tumor. The bone window in Figure 3 depicts the bony destructive nature of the mass. Fungal sinusitis was the first possible diagnosis. However, the differential diagnosis included nasal squamous cell carcinoma vs. sarcoma.

**Figure 4:**
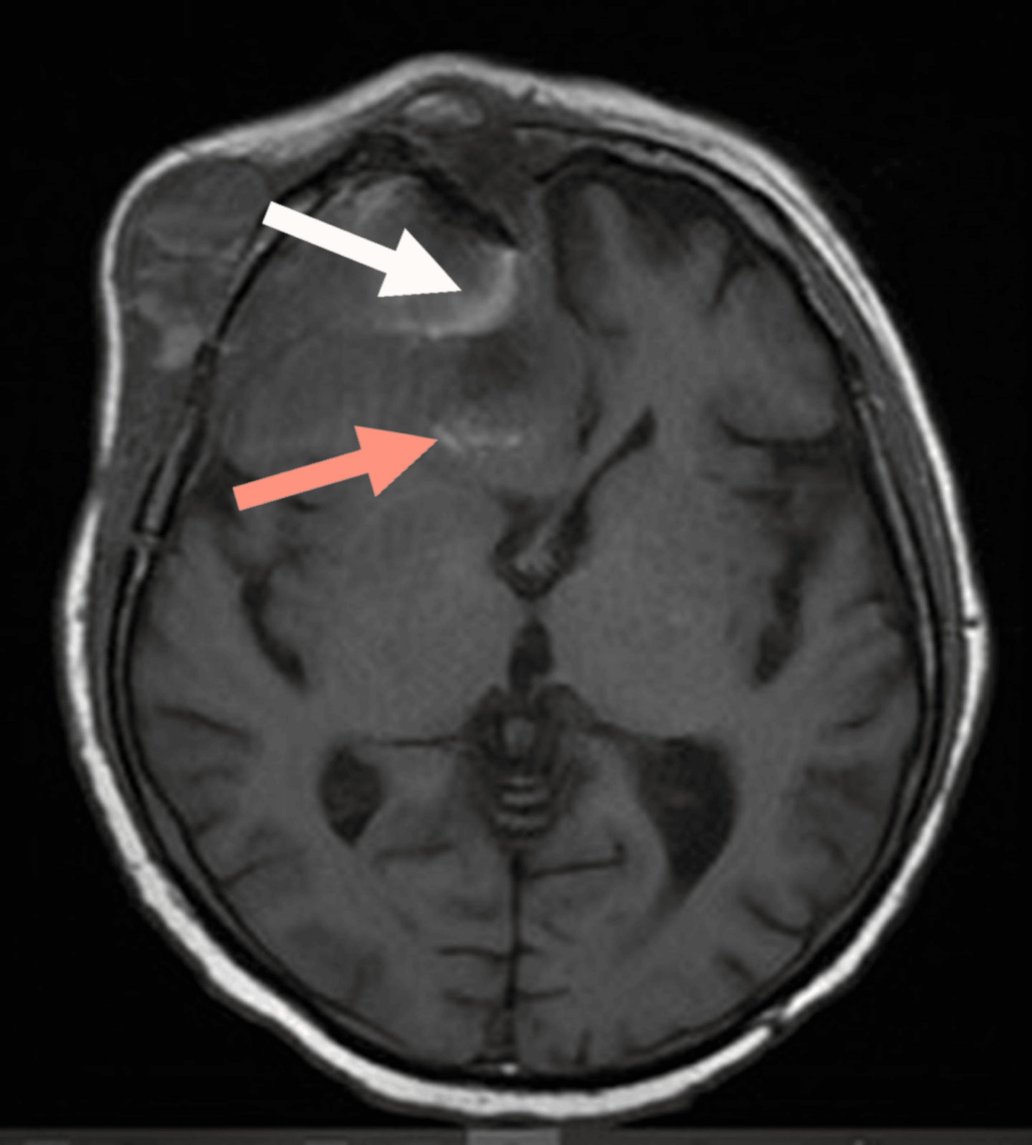
Axial T2-weighted MRI showing bright signal intensity within the mass (white arrow), with significant mass effect on the right lateral ventricle (red arrow).

**Figure 5:**
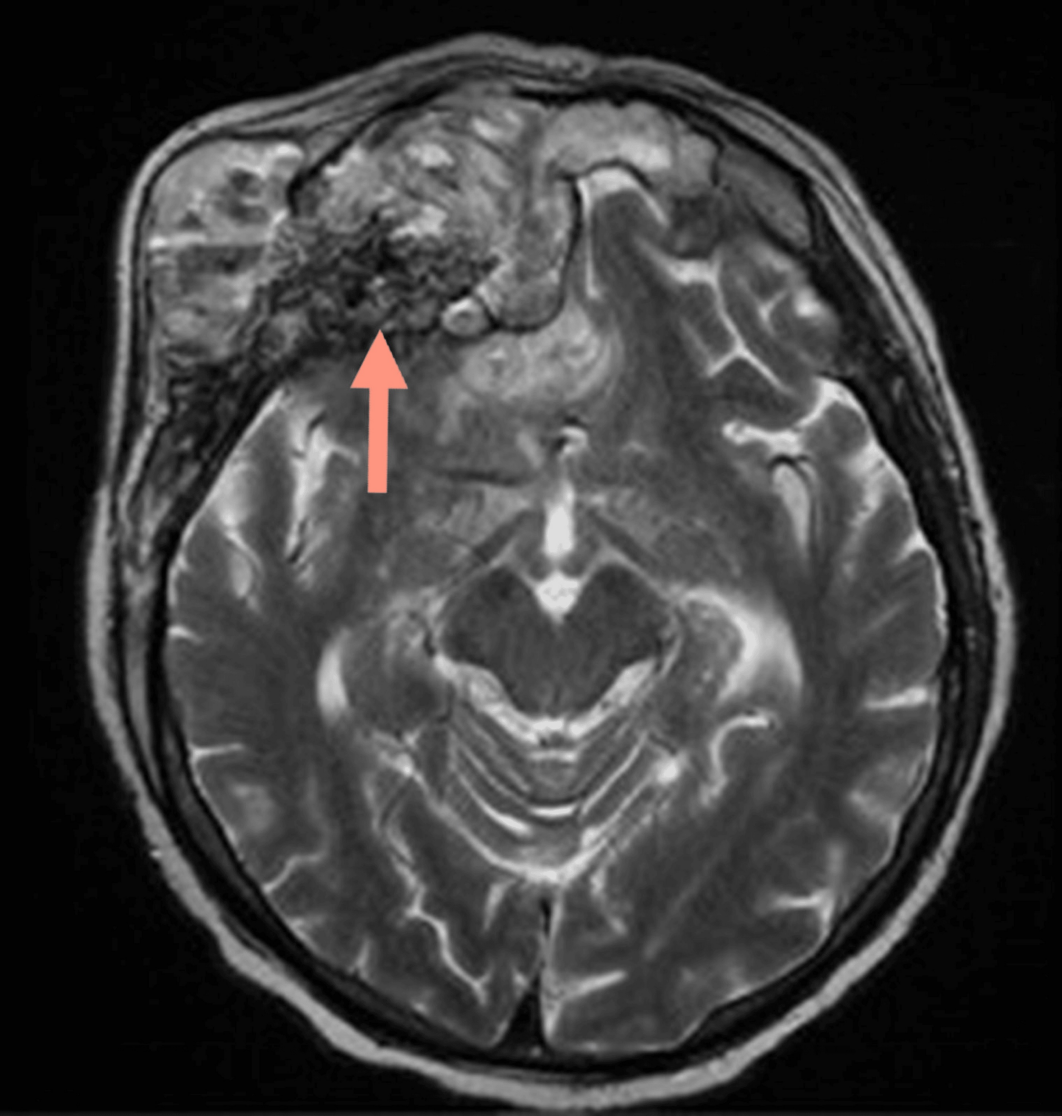
Axial T2-weighted imaging showing dark signal within the mass (red arrow).

**Figure 6:**
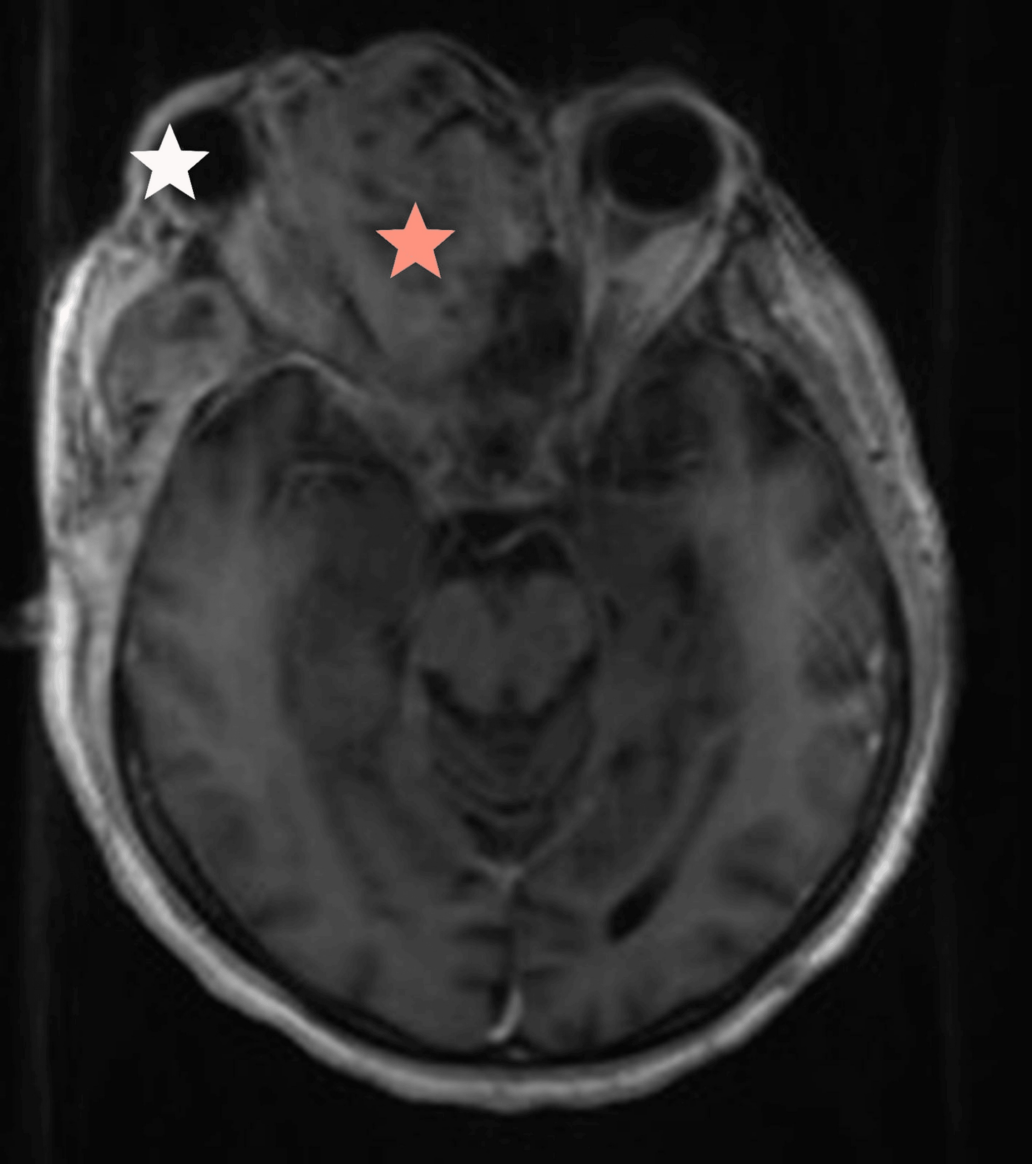
Axial T1-weighted MRI post contrast shows enhancement of the lesion (red star) with clear mass effect on the right orbital structures and proptosis (white star).

**Figure 7:**
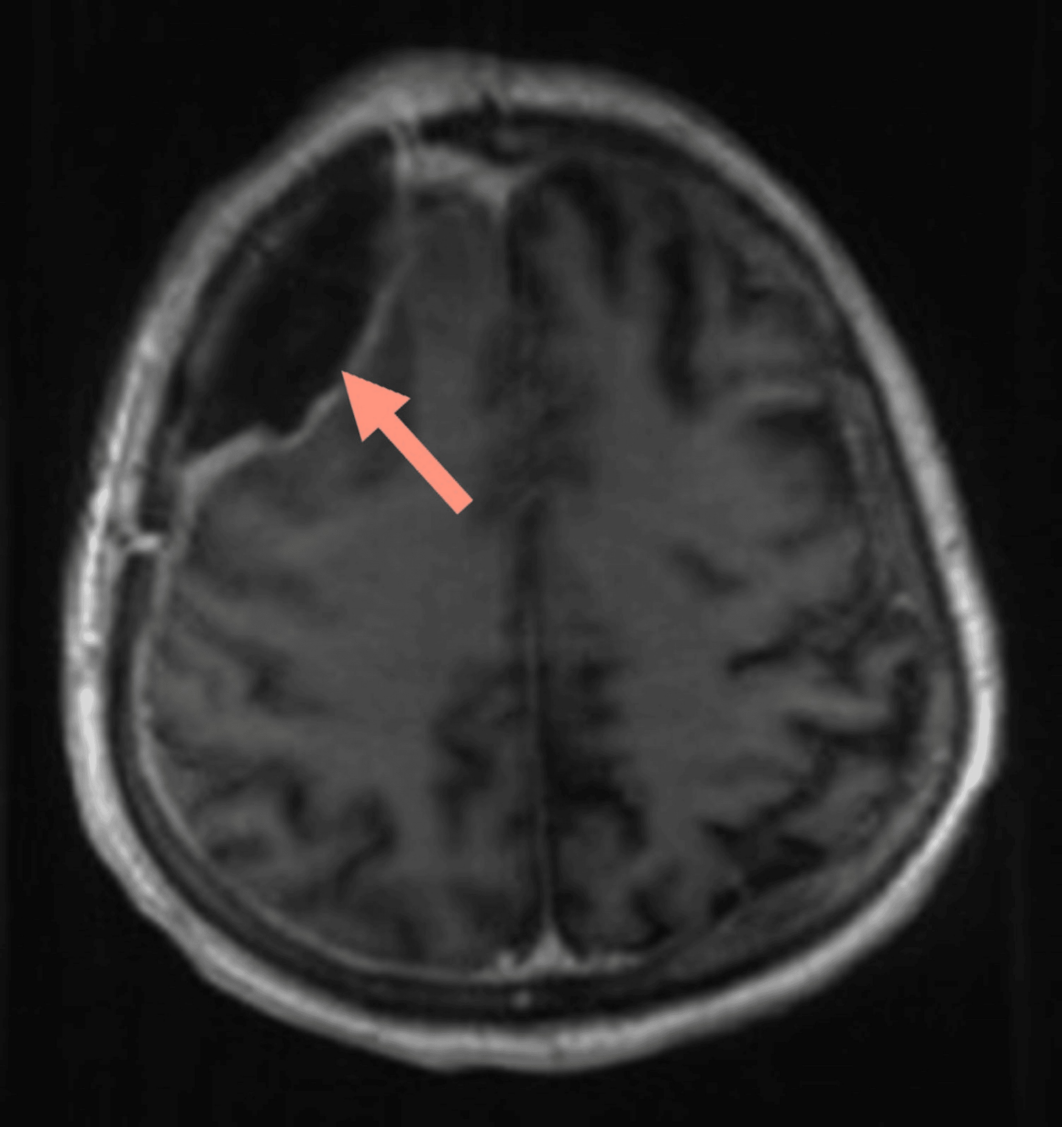
Axial T1-weighted MRI post contrast shows dural enhancement of the intracranial extra-axial extension of the tumor (red arrow).

CT was followed by an MRI with contrast of the brain, sinuses, and orbit for better delineation of the tumor. Findings were in keeping with aggressive mass lesion of the right nasal cavity, right ethmoid, and bilateral frontal sinuses with focal dural and cortical right frontal lobe invasion and significant mass effect and infiltration of the right orbit, as shown in Figures 4-7. Few areas of dark T2 and bright T1-weighted intensity were seen within the tumor, which were in favor of the diagnosis of melanoma rather than fungal infection.

**Figure 8:**
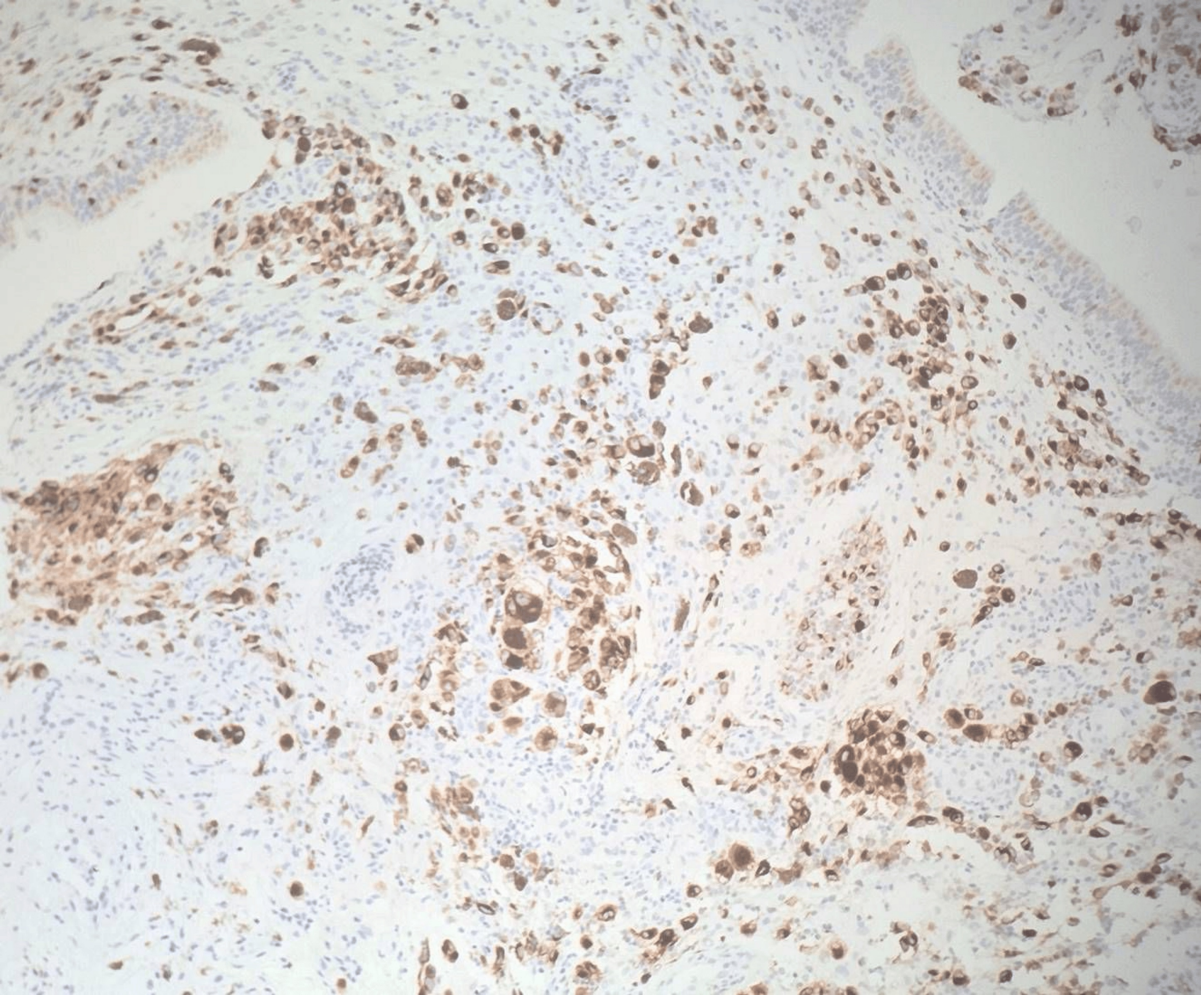
Hematoxylin & eosin stain (x10): Melanoma underlying the respiratory mucosa of the sinonasal area.

**Figure 9:**
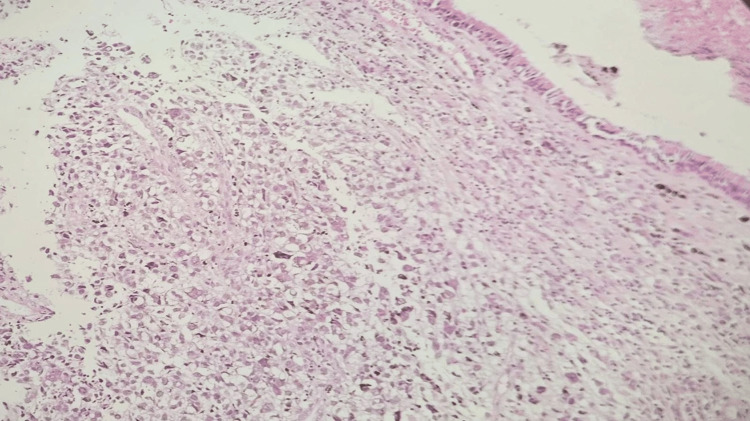
Hematoxylin & eosin stain (x20): Melanoma with prominent atypia, mitosis, and melanin pigments.

**Figure 10:**
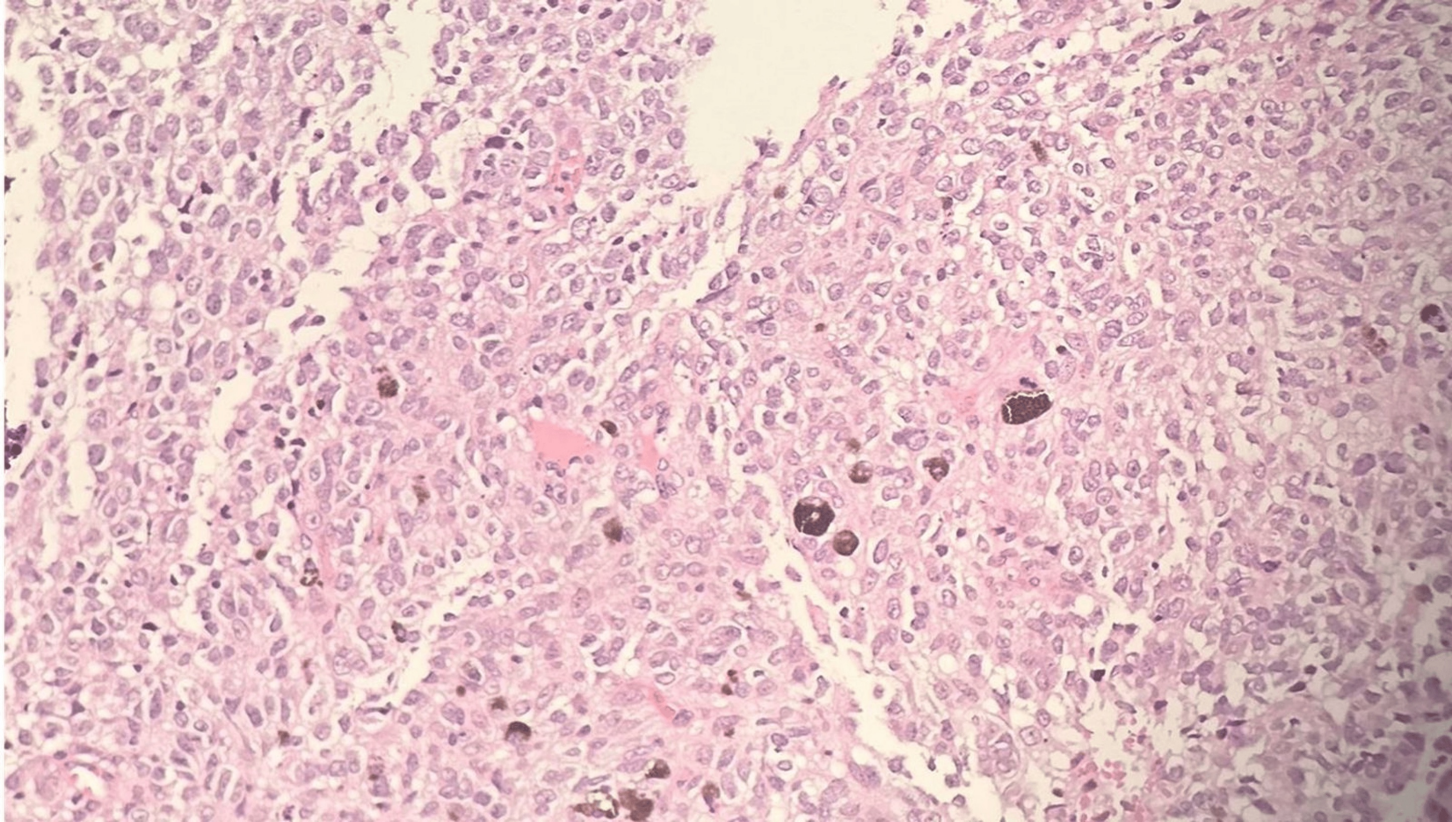
Immunohistochemistry stain, HMB45 (x10): Positive staining of HMB45 in melanoma.

Biopsy showed mucosal (sinonasal) melanoma. HMB45, S100, and Melan A were positive. CD45 (LCA), CKPAN, desmin, and MyoD1 were negative. Periodic acid-Schiff (PAS) stains were negative for fungal organisms. Detailed histopathology results are shown in Figures 8-10.

Local excision of the intranasal lesion under general anesthesia was performed. Unfortunately, the patient passed away one week after the operation.

## Discussion

Malignant melanoma of the paranasal sinuses is a rare type of malignancy that mostly affects male patients above the age of 70 years, which is typical for the current case. Nonspecific upper respiratory airway symptoms are usually the presenting features of this tumor. Our patient complained of unilateral nasal obstruction and epistaxis, in addition to headaches. Symptoms were progressive and did not respond to over-the-counter analgesics. Physical examination showed a right nasal cavity fungating mass with fragile mucosa.

Patients with suspected SNMM should be subjected to complete physical and ophthalmologic examinations to exclude local invasion and affection of the adjacent structures, i.e., orbits, in addition to enlarged metastatic cervical lymph nodes. Due to the aggressive course of the tumor, invasion of the adjacent structures is a common feature, so imaging is crucial to study the exact size and extension of the primary tumor, and to exclude local and distant metastasis for proper staging prior to treatment [1].

CT and MRI are important for the diagnosis and assessment of SNMM. CT is the recommended imaging modality of choice in cases of epistaxis and sinus-related symptoms. It usually gives a good idea about the size, location, and extent of the tumor and associated bone destruction, in addition to cervical lymph node metastasis [15].

In the current case, CT showed an invasive mass within the right nasal cavity associated with an antrochoanal polyp and sinusitis. Due to the presence of bony wall destruction and mass lesion, invasive fungal infection was the first possible diagnosis, followed by paranasal carcinoma.

The patient was referred to MRI for further characterization of the lesion. Characteristic MRI features may be detected in some cases, including foci of high T1 and low T2 signal intensities within the tumor. These specific signals may not be detected due to poor tumor differentiation or the presence of intra-tumor hemorrhage [15]. In the current case, findings were typical, where the mass was occupying the right nasal cavity, right ethmoid air cells, and frontal sinuses with typical high T1 and low T2 and fluid-attenuated inversion recovery (FLAIR) signal areas within the lesion. MRI was superior to CT in detecting the displacement of the adjacent recti muscles and right optic nerve.

A biopsy was requested to confirm the diagnosis before the treatment plan. As mentioned by Foreman et al., the diagnosis of SNMM rests on the identification of an atypical proliferation of cells with melanocytic differentiation. Melanocytic differentiation is established through immunohistochemical analysis and/or demonstration of melanin production by the tumor cells. The tumor is present in the mucosal epithelium and/or the submucosa. Evidence of metastasis from a cutaneous or ocular primary tumor must be excluded [1].

In the current case, typical findings of sinonasal melanoma were histologically detected, and at the same time, PAS stains were negative for fungal organisms.

In a study by Alves et al. [6], supported by many studies, the authors mentioned that available data from a pooled analysis of clinical trials indicate that efficacy outcomes seem to be poorer in mucosal melanoma compared to cutaneous malignant melanoma (CMM), with lower response rates and shorter survival.

## Conclusions

Sinonasal melanoma is a rare mucosal malignancy that usually affects older males more than females. It is usually diagnosed at a late stage due to its nonspecific upper respiratory tract symptoms, e.g., long-term nasal obstruction and epistaxis. Over time, symptoms of local invasion of the tumor may appear, including proptosis, diplopia, and changes in facial features. The tumor arises from the melanocytes lining the nasal cavity and paranasal sinuses, in addition to the mucosa of the genital system, lower rectum, anal canal, and other sites. Physical examination usually detects a fragile nasal mass. Ophthalmology examination can detect changes related to the local invasion of the tumor into the orbital structures. CT and MRI are essential for the diagnosis and staging of the tumor. Owing to its aggressive behavior and associated bone destruction, it is usually misdiagnosed as fungal sinusitis by CT. MRI shows characteristic findings of SNMM in the form of high T1 and low T2-weighted signal foci within the mass. However, a biopsy is mandatory to reach a proper diagnosis and to exclude non-sinonasal melanoma and fungal sinusitis. Excision of the tumor, followed by radiotherapy, is the main line of treatment. Owing to late diagnosis and frequent local and distant metastasis, the tumor has a poor prognosis and a high mortality rate.
